# Some of the Surgical Accidents of Childbirth1Read at the Thirty-sixth Annual Meeting of the Medical Association of Central York, at Buffalo, October, 1905.

**Published:** 1906-02

**Authors:** Willis E. Ford

**Affiliations:** Utica, N. Y.


### Some of the Surgical Accidents of Childbirth.[1]

BY WILLIS E. FORD. M. D., Utica, N. Y.

WHEN J. Marion Sims made a departure from general surgery that established a new specialty, gynecology, it was to repair surgical accidents of childbirth. Other men, not-

1. Read at the Thirty-sixth Annual Meeting of the Medical Association of Central York, at Buffalo, October, 1905.

ably, Emmet, and Peaslee, developing this work and departing into new fields which these brilliant men made visible, soon established a specialty which has flourished mainly because of the surgical accidents of childbirth. What obstetrics did in making gynecology possible, gynecologists studying the accidents of childbirth from a purely surgical standpoint, have more than repaid in the knowledge and prevention and the cure of those ailments which general physicians had not successfully managed. While abdominal surgery is chiefly the outgrowth of the investigations, skill, and patient experiments of gynecologists, the great bulk of this work is made necessary by the accidents of childbirth.

Forty years ago the cry in the profession was "Avoid meddlesome midwifery." So great was the strength of this sentiment that even religion was called to the aid of the waiting, hesitating accoucheur, and the statement that "In sorrow thou shalt bring forth children," was made to justify the delays which eventuated in accidents in a large number of cases. It was even thought wrong to give anesthetics. The surgical errors attendant upon childbirth were made known by the repairs which the gynecologists had to do afterwards. The prevention of sepsis was also in a large part the result of the same investigations, but methods were formulated and put in practice in a rational manner, as applied to obstetrics, by Garrigues of New York within the memory of most of those present. No historical statement would be correct without mentioning the name of Oliver Wendell Holmes, among those who have contributed much toward making childbearing so safe as it now is. There will always be certain surgical accidents and they may be enumerated as obstructed deliveries, hemorrhage, traumatism, and sepsis. Puerperal convulsions may not be surgical accidents, and yet they are considered as surgical by a large number of influential men of our profession. I can illustrate so many points in the surgical accidents of childbirth by one case, that I shall venture to relate it in detail.

Three years ago I was called to a neighboring town to repair the injuries done to a woman in childbirth. She had been delivered at noon of that day, after a labor which had been more or less active for three days. It was the first labor. The environment was such that it was not possible to believe that anything very aseptic could have been secured. The child was alive and weighed about eight and one-half pounds, and was delivered by forceps. The attending physician was a man of experience and skill, who said that he had been unable to deliver early in the morning, and that the head was not in the lower strait when the forceps were applied, and that the condition of the woman was so urgent that he had called assistance. He had administered chloroform and about noon, after very serious attempts at delivery, there seemed to be a sudden giving way of the resistance and the child was born. The hemorrhage was so terrific that the woman fainted after the birth of the secundines, and a hasty examination revealed the fact that she had been torn upwards, that the ramus of the pubis had been broken, and that the bladder and several knuckles of intestine were protruding. Without delay he took a large amount of iodoform gauze and pushed these viscera back, filling the vagina and packing as hard as he could. He succeeded in arresting the flow of blood. The woman was quite stout, weighing about one hundred and forty pounds. She was perfectly conscious when I got there, though very white and so weak she could hardly speak. Her pulse was weak and flickering at the wrist but her heart was sound. There was a little edema about the legs and ankles, which was said to have been present some weeks before delivery. Nothing wrong had been discovered with the urine.

After prolonged irrigation and scrubbing with green soap and water, and with the assistance of a nurse, while the doctor gave chloroform I carefully removed the packing and found that the ramus of the pubis was fractured one inch to the left of the symphysis, and that the bone surface was ragged and separated to the extent of about one and one-half inches. From above the cleft of the vulva at the left side just over the external ring, extending downward through the vagina was a tear. Not being prepared for it I was unable to wire the bones but brought them together by a stiff catgut placed in the periosteum on each side, and put on a tight bandage. I then began from above and closed the wound keeping the intestines and bladder back until I came down into the vagina. Here I found that the urethra had been severed its entire length, though still attached to the bladder, not cut crosswise, lying on the posterior wall of the vagina. I brought this forward and stitched it in place, and passing a catheter found that the bladder wall was intact, and then sewed an extensive rent in the posterior wall of the vagina, which went halfway up to the cervical junction. It was down to the sphincter ani but not through it. It had been discovered that the left ileum was freely movable, and I suspected a fracture at the site of the ileo-sacral union. Saline infusions, continuous stimulation and good nursing succeeded in bringing this woman through the first four days of her peril. She had no peritonitis at any time, the bowels moved naturally and the water was passed through the urethra.

On the fourth day she had a rise of temperature that was considerable, and soon after the water began to discharge freely through the vagina. Sloughing had taken place in the anterior wall of the bladder, due to prolonged pressure and traumatism. In about three weeks a large-sized abscess had developed in the left thigh just below the gluteal fold. She was then brought into the hospital and this abscess was opened and was found to communicate with some diseased bone at the ileo-sacral junction. She was septic at that time but soon recovered after thorough drainage and irrigation, and on examination was found to have sloughed more than one half of the anterior wall of the bladder, so that a vaginal speculum introduced in the ordinary way gave a perfect view of the posterior wall of the bladder and the left ureteral opening. There was some necrosis on the edges of the bone, and a fistula was then discharging into the top of the vagina. In fact, the front and roof of the bladder were gone. I curetted the dead bone, tightened the bandage about the pelvis, and got the ends of the bone in fair apposition, curetted away the cicatricial tissue, and brought the side of the bladder over and closed it, stitching it fast to the side wall. The urethra was intact. I fortified this row of silk interrupted sutures by continuous catgut to take off the tension, and the bladder held for two or three days, but the urine then began to leak. The opening at the top of the bladder over the point where the fracture had occurred was the most difficult to repair. The external conjugate was about seventeen centimeters, as against twenty and one-half; between the superior spines it was twnty-six, as against twenty-nine centimeters, and the anterior spines were twenty-one as against twenty-six centimeters. This showed the dystocia which made normal delivery and a normal birth impossible.

After three plastic operations, not counting the first procedure, I got down to a pin-hole opening at the junction of the cervix with the vagina. The woman would not remain for the fourth operation. She went home able to walk, with a perfect union at the seat of the fractured bone, with a small bladder but able to hold two or three ounces, and a year ago was reported by her physician as doing her work, and he says the fistulous opening has closed. I have not been able to find a report of a similar case. During this last week I saw this woman and she told me that she had borne another child since then with safety, though she had more leakage of urine. On examination I find that the bladder is torn in its anterior wall, and that the fistulous opening is large enough to admit the index finger. She has scarcely any exostosis about the site of the fracture, and is perfectly comfortable, and says that with the exception of the leaking of urine she would be in perfect health.

Taking the division of surgical accidents, hemorrhage, this case illustrates the fact that where extensive oozing surfaces give such a loss of blood as to endanger the patient, and the blood vessels concerned cannot be caught and tied, the ordinary rule of abdominal surgery and of general surgery would apply, that pressure with gauze is a safe thing to do.

Post partum hemorrhage after the third stage of labor may be due to a rent in the genital canal, and extensive tears in the neck of the uterus, or to an actual rupture of the uterus itself, or to oozing of the surface from uncontracted uterine muscle. After hot water has been tried and the compression of the uterus over the abdomen has failed, then with a tenaculum in the neck of the uterus a search should be made for the bleeding point. If it is in the neck of the uterus, catgut sutures should be applied and tied; but if the hemorrhage is from the body of the uterus, sterile gauze should be packed into the cavity, and the hand used to hold the uterus down on to the gauze. Even if the uterus is ruptured, this is the proper method until aid can be secured, so that the more formidable operation of opening the abdomen and suturing the uterus can be done with safety. Unless the rupture is extensive and there has been an escape of something beside blood into the abdominal cavity, this packing will in many cases be all that is required. It is safer to depend on this than to open the abdomen hurridly and without proper assistance, or in the absence of an experienced abdominal surgeon.

Some years ago I saw a case of severe laceration that had occurred twelve hours before, and in examining for the purpose of making an accurate diagnosis my finger passed through a rent in the uterus and on to a knuckle of intestine. I tamponed this with gauze and it was all that was necessary to be done for the uterus, though I later repaired an extensive tear in the perineum. I think it may be fairly assumed that the concensus of opinion is against immediate repair of a torn cervix, unless the hemorrhage is so great that for this reason stitches must be taken at once. Every case of severe hemorrhage should be carefully examined for this possibility by drawing down the cervix with a volsellum, so that the angles of this wound can be seen; and if hemorrhage comes from this wound, catgut sutures should be applied at once. If the hemorrhage is from the torn wall of the vagina and the hot water does not stop it, a continuous suture should extend from the upper end of the rent to the vaginal outlet. This is the common operation for torn perineum. The immediate repair of tears in the vaginal wall or perineum is imperative.

Absolute sepsis must be observed as far as possible the same as in the secondary operation for torn perineum. The stitches must be set deeply enough to leave no dead space beneath, and must not be drawn tightly enough to strangulate the tissues. I

think the common fault is in placing these stitches too superficially, and putting too much tension upon them when they are tied.

The needle that is used ought to be curved and long enough to extend to the bottom of the wound, and across and up out of the wound again.

When the rectum is torn through, after very careful irrigation the edges of the mucous membrance should be inverted into the rectum, and with fine catgut, taking great care as to the apposition of the parts, the entire length of the wound should be closed in the rectum with interrupted sutures about one-half an inch apart. The vaginal wall should then be carefully repaired, every other stitch catching part of the tissue just sewed, but not entering the rectum. The difficulty here I think is that generally the rectum is left too large, and the levator ani muscle is not caught in the upper tier of sutures. After the vaginal repair, iodoform gauze may be laid over the row of sutures, and replaced as often as it becomes soiled by the uterine discharges.

No discussion of hemorrhage in this connection would be complete without reference to placenta previa. Sound surgical principles applied here give the only promise of safety. The bleeding vessels cannot be caught and ligated, and hence pressure is the only resort. This is usually exerted by the hand of the operator while dilating so as to catch some part of the child and bring it down and use it as a plug. Tight gauze tamponing of the neck of the uterus may be made if the attachment be marginal or unilateral, but the hand is safer in most instances. Rigid asepsis is as important as is promptness in operating, and hence such patients should be taken to a hospital in all cases where it is possible to do so.

In addition to the tears due to traumatism there may be a separation of the fixed attachment of the bladder, so that eversion wholly or partially of the bladder and urethra may occur. This may not be repaired at the time but I think when such ectropion is marked, the parts should be carefully replaced and held by gauze tampons, through which a glass drainage tube may be fixed to carry off the lochia. Obstructed deliveries may result from several causes but chiefly from small or deformed pelves. Take the case I have just related: If she had been observed before labor and the deformed pelvis discovered, caesarian section would have been much more safe for her and the child than what she went through. Recent reports of successful cases of this operation seems to be better than symphyseotomy. The new operation, hebotomy,[1] appeals to me strongly as a rational measure, though I have never seen it done. It consists of severing the ramus of the pubis in exactly the place where this case of mine had the fracture. A thin saw can easily be passed beneath the bone and the pelvis separated. It is claimed that no after treatment is necessary, and my case would seem to justify the statement.

Ought not every physician, therefore, to impress on the minds of his clients the danger of such delay, for this poor woman called a physician only when in the pains of childbirth, and then insist on examining his patient early in her pregnancy.

If fibroid or other tumors are found which may endanger the delivery, the time to determine what must be done is early in pregnancy. The terrors of pregnancy, when fibroid tumors of the uterus are found, have diminished much since rigid aseptic methods have been generally followed. Women are delivered safely with almost any kind of tumor present unless it blocks the pelvic outlet. In this case, serious surgical interference is occasionally necessary, even to the removal of the tumor before labor is near. Sepsis does not frequently occur if rigid surgical rules are followed. The difficulty is that few men are sure of asepsis unless they have had some surgical training. If the nurse prepares the patient properly by scrubbing the external parts with green soap and hot water, and rinses with bichloride solution, a sterile napkin must be kept over the parts all the time afterward. If the physician's hands are carefully sterilised and enclosed in clean rubber gloves, he must not touch the clothing, or bedding, or his own person before an examination or an operation. It is perhaps harder to keep clean than it is to get clean.

If infection has occurred the most dangerous thing next is the sharp curet. With this there may be brought away considerable detritus, but much of this is nature's protecting film, intended to cut off the sinuses from putrefactive clots, or perhaps, fragments of membrane or placenta. A very dull curet may be used carefully to remove these fragments or to see if they remain, but this must be done with skill. Irrigation is useful and an ice coil to the abdomen, but too much surgery is bad. Anti-streptococcus serum is not uniformily useful, while hysterectomy is generally fatal. Isolating the uterus with gauze passed in through a vaginal incision has not met the expectations of those who have tried it. Aside from the douches and drainage, the case is medical practically, but the accumulation of pus in the pelvis which follows must be met with prompt incision and drainage.

While the causes that lead up to puerperal convulsions are medical and largely undetermined, the treatment is rapid delivery, and this is a surgical procedure. A good deal has been written lately about the futility of accouchment forcè, but I am convinced that the preponderance of opinion still favors it. The application of forceps in any condition should be looked upon as a surgical measure demanding asepsis of the parts operated, as well as of the instruments, hands, and dressings. The danger to the child is generally a reason for low forceps, and I am convinced that this is so real that to hesitate is often as bad as is meddlesome midwifery in its most offensive meaning. To prevent tears, hot compresses to the perineum and stretching this body between pains, is not observed so frequently in forceps cases as might be.

1 (*"Annals of Gynecology and Pediatry,"* October, 1904.)

266 Genesee Street.

